# Epigenetic Effects and Potential Contributions of m^6^A Modification to Mammary Gland Development and Lactation of Dairy Goats Explored via MeRIP-seq

**DOI:** 10.3390/ani15192775

**Published:** 2025-09-23

**Authors:** Lu Zhang, Zhibin Ji, Mingxin Zhao, Jianzhi Fu, Xianglei Meng

**Affiliations:** Key Laboratory of Efficient Utilization of Non-Grain Feed Resources, Shandong Provincial Key Laboratory for Livestock Germplasm Innovation & Utilization, College of Animal Science and Technology, Shandong Agricultural University, Ministry of Agriculture and Rural Affairs, Tai’an 271018, China; 18596258003@163.com (L.Z.); 17861822722@163.com (M.Z.); jianzhi2699@foxmail.com (J.F.); mxl20021121@163.com (X.M.)

**Keywords:** m^6^A, dairy goat, mammary gland, MeRIP-seq, RNA-seq

## Abstract

To date, more than 170 chemical modifications have been identified in RNA. m^6^A (N^6^-methyladenine) RNA methylation is the most abundant form of mRNA modification in eukaryotes, playing an important role in RNA post-transcriptional processes. To investigate the function of m^6^A methylation modification in the development and lactation of dairy goat mammary glands, mammary gland tissue samples were collected in the early (20 days postpartum), peak (90 days postpartum), and late period (210 days postpartum) of three dairy goats. MeRIP-seq and RNA-seq were used to explore m^6^A methylation modification events. We identified 1638 differential peaks in the MeRIP-seq data across 1539 differentially methylated genes, which were enriched in ribosome biogenesis in eukaryotes, Toll-like receptor signaling pathway, TNF signaling pathway, MAPK, and other pathways related to mammary gland development and lactation. A conjoint analysis revealed that 179 common differential expressed genes were obtained, of which 150 were negatively regulated by their m^6^A modifications, while 5 common differentially expressed genes—*PPARG*, *HSPA2*, *CDK5*, *ACTB* and *NOTCH3*, were screened out in the two groups. In conclusion, m^6^A modification involves the pathways related to mammary gland development and lactation by modifying gene expression. This studyprovides new insights into m^6^A epigenetic regulation, mammary epithelial gene networks, and actionable molecular targets for high-value dairy product production and the breeding of new varieties.

## Simple Summary

To investigate the role of m^6^A methylation modification in the development and lactation ability of dairy goat mammary glands, tissue samples were collected during the early period (20 days postpartum), peak (90 days postpartum), and late period (210 days postpartum. MeRIP-seq combined with RNA-seq was used to explore m^6^A methylation modification events. There were 1638 differential peaks in the MeRIP-seq data, across 1539 differentially methylated genes, which were enriched in the Toll-like receptor signaling pathway, MAPK and other pathways related to mammary gland development and lactation. A conjoint analysis of RNA-seq and MeRIP-seq revealed that, among the 179 common DEGs identified, 150 genes exhibited negative regulation as a results of m^6^A modifications. Five key DEGs—*PPARG*, *HSPA2*, *CDK5*, *ACTB*, and *NOTCH3*—were consistently dysregulated in both groups. In summary, m^6^A modification involves pathways related to mammary gland development and lactation in ways that are gene and context specific. This study provides new insights into the physiological and epigenetic regulation mechanisms of mammary gland development and lactation in dairy goats.

## 1. Introduction

Lactation biology in ruminants is crucial for both animal production and agricultural economics. Understanding the physiological, biochemical, and genetic aspects of lactation helps to improve milk yield, quality, and overall herd health. Adequate dairy farm managementcannot be achieved without understanding the biological mechanisms behind lactation in ruminants [1,2]. Mammary gland lactation biology is a complex physiological process that undergoes a cycle of proliferation, differentiation, lactation, degeneration, apoptosis, and mammary gland tissue remodeling [3]. These provesses are comprehensively regulated by various factors, including neurohormones; material metabolism; the number and activity of mammary gland epithelial cells and gene expression and signal transduction [4,5].

N6-methyladenosine (m^6^A) is the most abundant internal modification of RNA molecules in eukaryotes, which plays a crucial role in post-transcriptional gene regulation [6]. m^6^A is methylated at the sixth N position of adenylate, and is mainly enriched in the 3′UTR region, stop codon region, and specific motif sequences (RRACH, DRACH) [7]. m^6^A modification is a dynamic and reversible process that relies on methylases and demethylases [8]. Methylases add m^6^A modification to mRNA, whereas demethylase can reverse the effect of m^6^A methyltransferase and remove m^6^A modification. Growth factors such as *IGF2BP1*, *IGF2BP2*, and *IGF2BP3* can recognize RNA methylation information, participate in downstream translation and mRNA degradation, and can accelerate mRNA export [9,10]. Recent studies have suggested that m^6^A methylation influences mammary gland development, milk synthesis, and lactation efficiency in ruminants by regulating mRNA stability, including localization, transport, alternative splicing, degradation and translation [11].

Thus far, research on m^6^A methylation modification in the breast has mainly focused on breast cancer [12,13]. In domestic animals, researchers have only studied such mechanisms in goat, cow, pig, chicken, and goose, considering factors such as early embryonic development, secondary hair follicle development [14,15,16], adipocyte differentiation [17,18], muscle growth and development [19,20,21], milk synthesis in mammary epithelial cells [22], and gonadal differentiation of chicken [23]. For example Wang et al.’s (2024) researches in vitro GMECs indicated that m^6^A methylation mediates the function of the circRNA-08436/miR-195/*ELOVL6* axis with regard to lipid metabolism in dairy goat mammary glands [24]. Qi et al. (2022), revealed at the transcriptome-wide level, that the alteration of N6-methyladenosine modification played a key role in the heat stress response in bovine mammary epithelial cells by regulating gene expression [25].

Although m^6^A has been profiled in bovine and murine mammary cells, its dynamics across distinct lactation stages in dairy goats have yet to be determined. In this study, transcriptome-wide m^6^A peaks in the early, peak, and late lactation period were mapped using methylated RNA immunoprecipitation sequencing (MeRIP-seq), which was further integrated with RNA-seq to identify co-regulated genes, The characteristics of core m^6^A regulators and functional pathways in goat mammary tissue were identified in different stages of the mammary gland development and lactation of dairy goats. This study may provide a new theoretical basis for deep research on the physiological epigenetic regulation mechanisms of mammary gland development and lactation in dairy goats. We hypothesized that stage-specific m^6^A remodeling underlies shifts in lipid metabolism and epithelial proliferation.

## 2. Materials and Methods

### 2.1. Experimental Samples

Three healthy Laoshan dairy goats were randomly selected from the Qingdao Aote Dairy Goat Farm. The goats were of a variety cultivated in Shandong province in China. All animals were one year old, with similar first parity, similar physiological status, no history of mastitis, and a similar body condition score, and were raised and managed in the same way. All dairy goats were sacrificed without pain after being administered general anesthesia via intramuscular injection of xylazine hydrochloride injection solution (100 ug/mL; 0.3 milligrams per kilogram of body weight). Every effort was made to reduce the suffering of the animals involved in the experiments We collected nine mammary gland tissue samples (*n* = 3 per stage) located in the posterior mammary region near the groin, in the early lactation (20 days postpartum), peak lactation (90 days postpartum), and late lactation (210 days postpartum) stages. The collected mammary gland tissues were quickly placed in a centrifuge tube, and immediately put into a liquid nitrogen tank for storage. All animal experimental procedures in this study were conducted in accordance with the Animal Experiment Management and Use Committee of Shandong Agricultural University (SDAUA-2021-130, Table S7).

### 2.2. RNA Extraction and Library Construction

The mammary gland tissues were transferred to a mortar. A small amount of liquid nitrogen was added and the mixture was quickly ground into powder, then placed in a centrifuge tube. Total RNA was extracted using a Trizol kit (Catalog number: 15596026, Invitrogen, Carlsbad, CA, USA), according to the manufacturer’s instructions. TheRNA quality, concentration, and integrity were tested via an Agilent 2100 Bioanalyzer (Agilent Technologies, Santa Clara, CA, USA). Only RNA samples with an integrity number (RIN) greater than 6.5 were selected for subsequent analysis. A gel electrophoresis instrument (Agilent Technologies, USA) with RNase-free agarose gelation was also used to detect the quality of the total RNA.

After the extraction of the total RNA, 1 mg of poly A-tailed total RNA was enriched and purified using Oligo (dT) beads with Protein G (NEB, Ipswich, MA, USA, Category number: S1430), and the binding reaction was performed at room temperature for 5 min with continuous mixing. Magnesium fragmentation buffer (Ambion, Austin, TX, USA) was used to convert the enriched mRNAs into approximately 100 nt fragments. Then the mRNAs were divided into two parts. Firstly, 10% of the RNA was used for initial quality control (input, RNA-Seq), and to directly construct the transcriptome libraries. The remaining 90% was enriched with m^6^A-specific antibodies (incubated for two hours at 4 °C), and purified RNA fragments were eluted in IP buffer. Two copies of mRNA were reverse transcribed into cDNA using the NEBNext^®^ Ultra™ II RNA Library Prep Kit for Illumina^®^ (Catalog number: E7770L, NEB, USA). The purified double-stranded cDNA fragment was end-repaired, poly A-tailed was added, and the fragment was conducted to the sequencing adapter. Then, cDNAs of approximately 200 bp was screened using AMPure XP Beads (NEB, USA) and, after PCR amplification, the PCR products were purified again using 1X AMPure XP Beads to construct the libraries (immunoprecipitation, MeRIP-Seq), and the quality of the libraries was tested using denaturing agarose gel electrophoresis. The sequencing and data collection were carried out using an Illumina Novaseq 6000 in Kidio Biotechnology Co., Ltd. (Guangzhou, China).

### 2.3. Quality Control and Sequence Comparison of Sequencing Data

The Fastp software (version 0.18.0) [26] was used to perform quality control on the raw reads (parameters: −q 20 −u 50 −n 15 −l 20). Reads containing adapters, reads containing a N ratio greater than 10%, and reads containing more than 50% low-quality bases (*q* value ≤ 20), were removed, then clean reads were obtained. The high-quality clean reads were aligned to the ribosome database of *Capra hircus* using the short-reads alignment tool bowtie2 software (version 2.2.8) [27], to remove the RNA reads aligned to ribosome database, and the remaining clean reads were used for alignment analysis of the *Capra hircus* reference genome (version: GCF_001704415.1_ARS1) using the HISAT2 software (version 2.2.4) [28].

### 2.4. m6A Peak Identification of Mammary Gland Tissue at Different Lactation Periods

A gene annotation file (https://www.ncbi.nlm.nih.gov/datasets/genome/GCF001704415.1, accessed on 1 June 2021) was used for peak identification. For MeRIP-seq data, the MACS2 software (version 2.1.2), with the parameter ‘–nomodel’ separately, was used to identify the enriched regions of m^6^A, and significant peaks with a *p*-value < 0.05 for specific regions were calculated based on dynamic Poisson distribution. The peaks in genomic positioning and gene annotation were scanned, analyzed, and identified. The homer software (version 4.10, MotifsGenome.pl {peak_file} {genome.fa} {out_dir}) was used to construct an averaged base frequency matrix for each motif for enrichment analysis and the frequency distribution of specific motifs (RRACH, DRACH) in all peaks was calculated. The DiffBind software (version 2.8) was used to merge the peaks between different groups to obtain all peaks for each treatment group. The peak abundance was displayed by calculating the rpm value (reads per million IP reads) of each sample. The RNA methylation rate (RPM (MeRIP)/RPM(input)) was used to filter the differential peaks between different groups (*p* value < 0.05 and |log2FC| > 1).

### 2.5. Functional Analysis of Differential Peaks Between Different Lactation Periods

The metscape online tool was used to map genes with differential peaks to GO (http://www.geneontology.org/, released 4 March 2016) and KEGG (https://www.genome.jp/kegg, Release 87.0, accessed on 3 March 2022) databases, to identify significantly enriched GO terms and pathways (*p* ≤ 0.05), to determine the functions of peak-related genes, as well as the most important biochemical metabolic pathways and signal transduction pathways.

### 2.6. Conjoint Analysis of RNA-seq and MeRIP-seq

Based on m^6^A-modified and non-m^6^A-modified genes, the Weishengxin online platform (http://www.bioinformatics.com.cn/) was used to visualize and analyze the impact of m^6^A modification on gene expression. The Wald test in the DESeq2 [29] software v1.26 was used for the differential expression analysis, and *p* < 0.05 and |log2FC| ≥ 1 were executed as the criteria. Briefly, DESeq2 was used to model the raw counts, using normalization factors (size factors) to account for differences in library depth. Then, the gene-wise dispersions were estimated and the estimates were shrunk to generate more accurate estimates of dispersion to model the counts. Finally, the negative binomial model was fitted and hypothesis was performed testing using the Wald test or the Likelihood Ratio Test. The Excel software (version 2016) was used to screen the common differential genes in two groups; the Origin Pro v8.6 software was used to draw a Venn diagram of the common differential genes; and Graphpad Prism v6.01 was used to draw a nine-quadrant diagram of the common differential genes (|log2FC| > 1), and analyze the co-regulation relationship of the common differential genes in two groups.

### 2.7. Identification of Core Genes and Construction of PPI Regulatory Networks

Based on the enrichment results of GO and KEGG, differentially methylated genes (DMGs) related to mammary gland development and lactation were screened, as well as common differential genes in RNA-seq and MeRIP-seq with a standard of |log2FC| > 1 and *p* ≤ 0.05. The potential interactions between genes in mammals were searched through the STRING database (v11.0) with a confidence score cutoff of more than 0.4. The CytoHubba plugin for the Cytoscape software (Version 3.10) was used to screen core genes based on a degree of more than 15, as well as the connectivity of each node, and CytoNCA pluging was used to draw an interaction network.

## 3. Results

### 3.1. Sequencing Data Quality Assessment and Reference Genome Alignment

**Figure 1:**
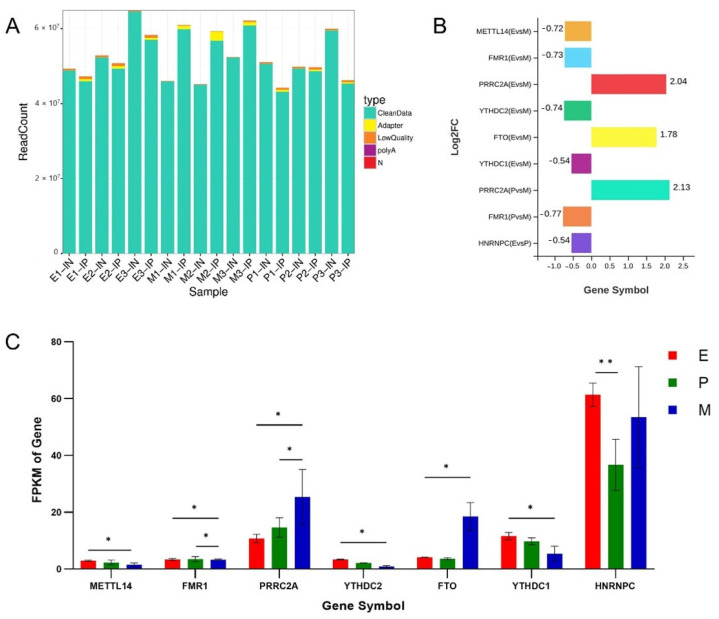
Sequencing data statistics and identification of m^6^A methylases. (**A**) Statistical graph of reads filtered information. (**B**) Plot of the multiplicity of m^6^A methylase differences between different groups. (**C**) The amount of m^6^A differentially methylated enzyme expression. Note: IN represents control group, IP represents MeRIP group, E represents early lactation, P represents peak lactation, and M represents the end of lactation; * represents *p* < 0.05 difference and ** represents *p* < 0.01 significant difference.

In this study, the mammary gland tissues of dairy goats during early lactation (E), peak lactation (P), and late lactation (M) were selected respectively for MeRIP-seq and RNA-seq, with three biological samples in each group of replicates. After raw data filtering, the distribution of high-quality data in each library was plotted, as shown in Figure 1A, for the MeRIP-seq groups (IP groups), and 50780598, 59149108, and 45723622 clean reads were identified in E, M, and P, respectively. Correspondingly, for the RNA-seq groups (IN groups), they were respectively distributed in quantities of 55231892, 47726652, and 53170566. The clean reads obtained from both sequencing groups accounted for more than 95.72% of the total reads. The base composition and quality distribution of each library were as shown in Table 1. Q20 of the data after quality control analysis at different lactation periods, for the MeRIP-seq groups, ranged from 92.21% to 98.25%, whereas Q30 ranged from 85.39% to 95.80%, and the GC content was more than 48.06%. Meanwhile, for the RNA-seq groups, Q20 was distributed in 97.26~97.65%, and Q30 showed distribution in 92.02~92.96%. Comparative analysis of clean reads of different lactation periods with the reference genome showed that, for the MeRIP-seq groups, the alignment rate of all reads that can be located on multiple loci in the genome showed a range of from 10.56~20.54%, while the alignment rate with one location was distributed in 54.89~78.13%. For the RNA-seq groups, they achieved ranges of 3.98~10.77%, and 85.69~91.72%, respectively (Table 1). Additionally, a comparison of the high-quality clean reads with the ribosome database indicated that less than 0.04~0.29% of the clean reads were aligned on the ribosome database, and this information was discarded in the subsequent analysis. The above results show that the sequencing quality of each library was high and the data were reliable, indicating that they were suitable for subsequent analysis.

### 3.2. Identification and Analysis of m6A Methylase

Through an analysis of the sequencing data, seven differential methylases were screened out according to *p* value < 0.05 and |log2FC| > 1 (Figure 1B,C, Supplementary Materials File Table S1). Furthermore, m^6^A methylases, METTL14, FMR1, PRRC2A, YTHDC1-2, and FTO— were differentially expressed in E vs. M. Of these METTL14, FMR1, PRRC2A, and YTHDC1-2 were highly expressed in the E period, while PRRC2A and FTO were highly expressed in the M period, and the multiple differences of PRRC2A and FTO were the largest between the two periods. PRRC2A and FMR1, were screened out in P vs. M, PRRC2A was highly expressed in the M period and FMR1 was highly expressed in the E period. There was only one differentially expressed gene in E vs. P, HNRNPC, which was upregulated in the P period compared with the E period. These methylase expression results indicated that m^6^A methylation modification levels vary in the different lactation periods of dairy goats.

### 3.3. m^6^A Peak Annotation and Motif Analysis

**Figure 2:**
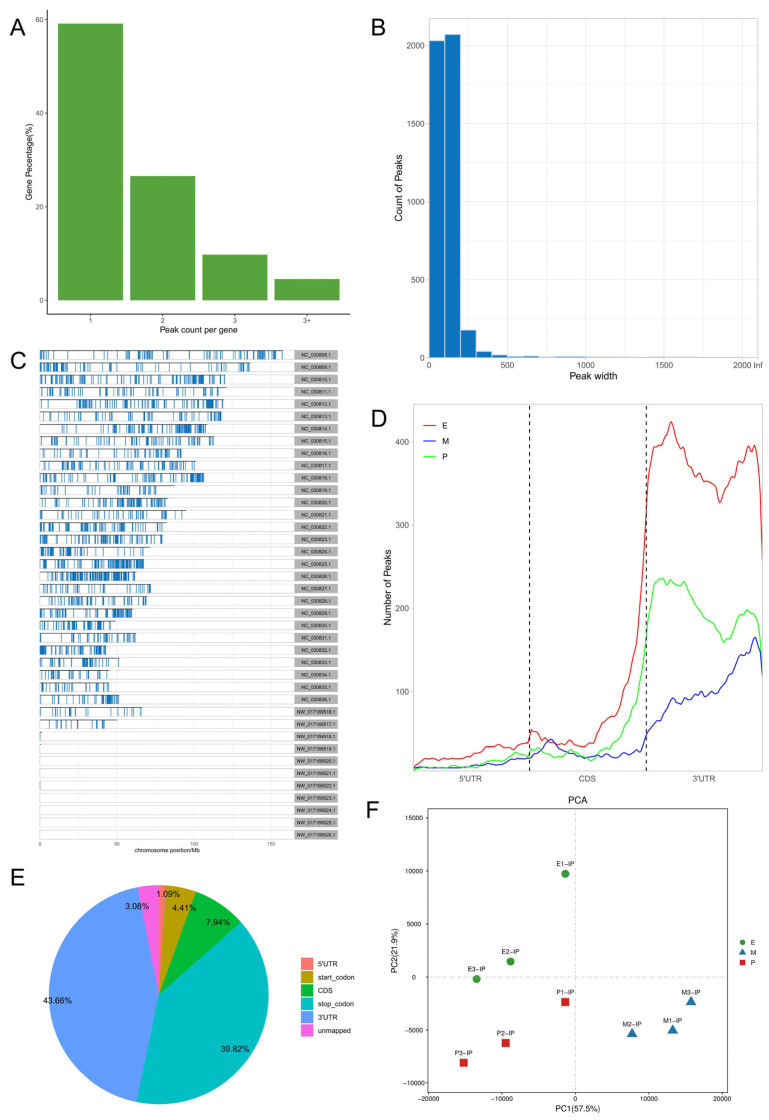
m^6^A peak annotation and motif analysis of mammary glands during different lactation periods. (**A**) Statistical map of the peaks in the number of genes. (**B**) Peak width distribution map. (**C**) Peak distribution of chromosomes. (**D**) Peak distribution in transcripts. (**E**) Distribution of peask in different functional elements. (**F**) Correlation heatmap of all IP samples.

For the m^6^A peak identification, 3338 3302, and 3368 were excited in three libraries for the E period, where as for the P period, this was observed for 3287 3176, and 3109, respectively. In the M period, 1684, 1249, and 896 peaks were obtained. After filtering and merging the peaks in different periods, 2476 952, and 1451 m^6^A peaks were detected in the E, P, and M periods, respectively. A total of 3332 peaks were detected in three lactation periods (Supplementary Materials File Table S2). By analyzing the m^6^A peak distribution of each gene, it was found that 59% of the genes showed one peak, and relatively few genes had two or three peaks (Figure 2A). The peak width was mainly concentrated within 200 bp (Figure 2B). The distribution of m^6^A peak on chromosomes is shown in Figure 2C. It is significantly distributed on 31 chromosomes, and widely and dispersedly distributed on chromosome NC_030808.1, while their distribution is small and concentrated on NC_030825.1 and NC_030826.1. The distribution of peaks on gene functional elements was mainly located in 3′UTR and the stop codon of the coding region (Figure 2D,E). A principal component analysis of the correlation of all samples found that the clustering trend of samples in the same period was consistent, there was better repeatability within the sample group, and there was good discrimination between different groups (Figure 2F). An averaged base frequency matrix was constructed for specific motifs (RRACH, DRACH) in all peaks, and it was also found that the most enriched m^6^A site sequences in different periods were AAACA, GGACT, and AAACA (Table 2).

### 3.4. GO Annotation and KEGG Enrichment of DMGs

**Figure 3:**
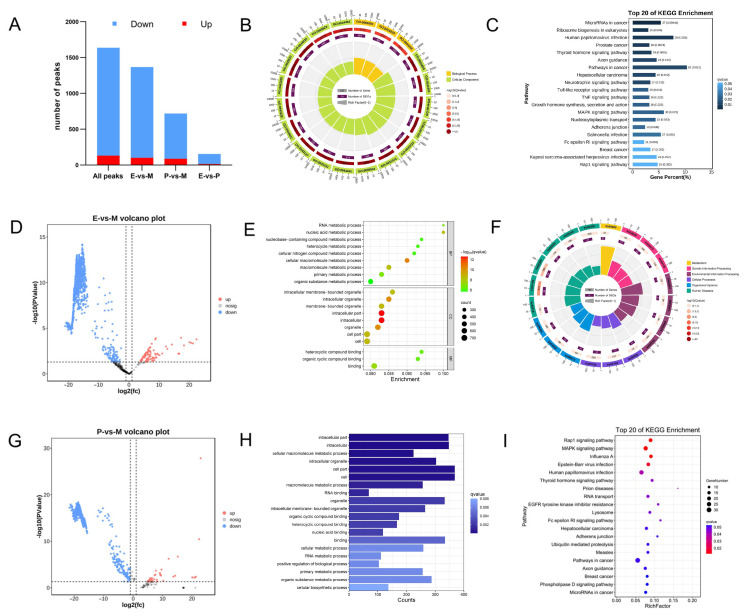
GO annotation and KEGG enrichment of differentially m^6^A methylated genes between different lactation periods. (**A**) Statistical map of differential peaks between different periods. (**B**) GO enrichment map of total differential methylation genes. (**C**) KEGG enrichment map of total differential methylation genes. (**D**) Volcano map of differential peaks between E-vs.-M periods; (**E**) GO enrichment map of differential peaks between E-vs.-M periods. (**F**) KEGG enrichment map of differential peaks between E-vs.-M periods. (**G**) Volcano map of differential peaks between P-vs.-M periods. (**H**) GO enrichment map of differential peaks between P-vs.-M periods. (**I**) KEGG enrichment map of differential peaks between P-vs.-M periods.

To further analyze the function in the mammary gland of m^6^A methylation genes, RNA methylation rate differences were analyzed for all peaks in all groups to screen the differential peaks (*p* < 0.05, |log2FC| > 1). The total number of differential peaks in three periods was 1638 across 1539 DMGs, of which 130 were upregulated and 1508 were downregulated (Figure 3A, Supplementary Materials File Table S3). The results of the GO analysis show that DMGs were distributed in 17 cell components, such as intracellular membrane-bound organelles, the cytoplasm, and the nucleus. 26 biological processes, which mainly included metabolic processes, single biological processes, and biological regulatory processes, and 13 molecular functions, including protein binding, enzyme binding, compound binding, and catalytic activity. (Figure 3B, Supplementary Materials File Table S3). KEGG was significantly enriched in 16 pathways, including axis guidance, ribosome biogenesis in eukaryotes, the Toll-like receptor signaling pathway, the TNF signaling pathway, the MAPK signaling pathway, and growth hormone synthesis, secretion, and functions (Figure 3C, Supplementary Materials File Table S3).

In the E vs. M period, 1367 differentially expressed peaks were identified, of which 101 were upregulated, involving 1156 DMGs, and 1266 were downregulated, involving 98 DMGs (Figure 3A,D). GO enrichment analysis found that DMGs were significantly enriched in 183 secondary GO terms, including 35 cellular components, 139 biological processes, and 9 molecular functions, which were mainly enriched in various metabolic processes and the binding of cells and organelles. (Figure 3E, Supplementary Materials File Table S3). KEGG was mainly enriched in RNA transport, Endocytosis, Hedgehog signaling pathway, ribosome biogenesis in eukaryotes, and the Wnt signaling pathway (Figure 3F, Supplementary Materials File Table S3).

In the P vs. M period, 719 differential peaks were found, of which 87 were upregulated and 632 were downregulated, including 710 DMGs (Figure 3A,G). GO analysis found that 710 DMGs were enriched in 86 secondary GO terms, including 9 cellular components, 72 biological processes, and 5 molecular functions, such as cells and organelles in cellular components, ribonucleic acid and cyclic conjugates in molecular functions, the positive regulation of various metabolic processes, and cell biosynthesis, among other biological processes (Figure 3H, Supplementary Materials File Table S3). KEGG analysis showed that DMGs were enriched in pathways related to cell growth, development, proliferation and apoptosis, such as the Rap1 signaling pathway, the MAPK signaling pathway, and the thyroid hormone signaling pathway, and RNA transport (Figure 3I).

### 3.5. Correlation Analysis of MeRIP-seq and RNA-seq

**Figure 4:**
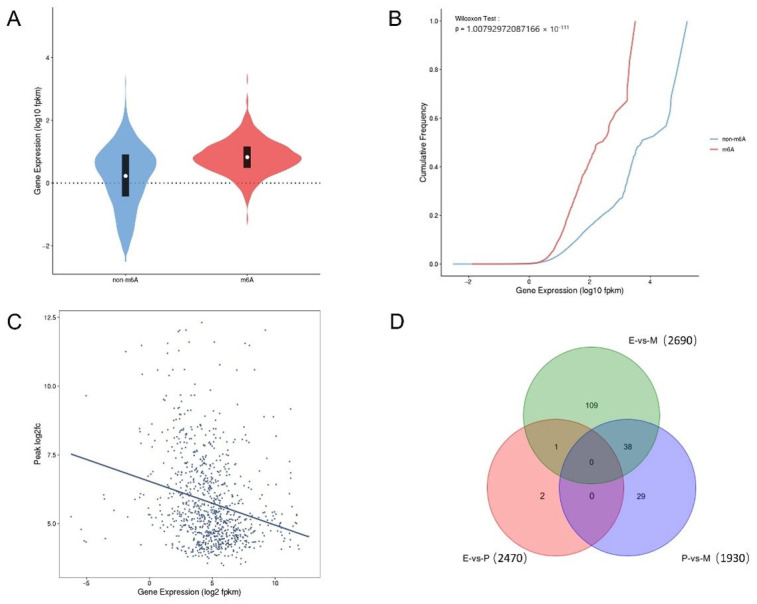
Statistical results of correlation data between the MeRIP group and RNA-seq group. (**A**) Violin plots of genes with/without m^6^A modification. (**B**) Expression of cumulative curves of genes with/without m^6^A modification. (**C**) Statistical plot of enrichment fold change in genes’ expression and peaks. (**D**) Venn diagram of common differential genes in the MeRIP group and RNA-seq group.

Judging from the overall expression level of genes, these genes were regulated in gene- and context-specific ways. Firstly, from the overall levels of genes in the present study, it can be seen that the expression level of genes with m^6^A modification was higher than that of genes without m^6^A modification, indicating a more frequently probability of m^6^A appearing in genes with high expression levels (Figure 4A). Secondly, from the cumulative curve chart, it can also be seen that at the same cumulative frequency, the corresponding curve was skewed to the left as the genes were modified by m^6^A methylation, indicating that the expression level of genes modified by m^6^A is lower, and m^6^A modification has the potential to negatively regulate gene expression (Figure 4B). Thirdly, from the statistical results of gene expression and multiple-peak enrichment, it can be seen that genes with a relatively high expression level have relatively low peak enrichment, indicating that m^6^A modification can also negatively regulate genes’ expression level (Figure 4C). The above results show that m^6^A modification is closely related to gene expression and can change the stability of mRNA, to a certain extent.

According to RNA-seq sequencing, a total of 21976 genes were obtained from dairy goat mammary gland samples at three different lactation periods, including 20606 known genes, 1370 new genes, and 3460 DEGs (Supplementary Materials File Table S4). In the joint data statistics of m^6^A-seq and RNA-seq, there were 179 DEGs between the different periods. In E vs. M, there were 2690 genes and 148 DEGs, while in P vs. M, there were 1930 genes and 67 DEGs (Figure 4D, Supplementary Materials File Table S5).

### 3.6. Co-Regulation Relationship of Common DEGs in MeRIP-seq and RNA-seq

**Figure 5:**
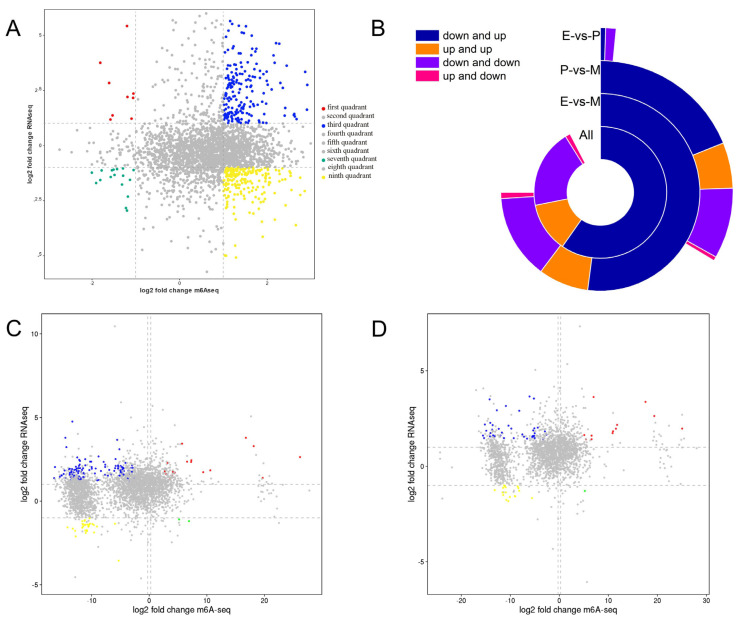
Co-regulatory relationships of common differential genes shared by MeRIP and RNA-seq group. (**A**) Nine-quadrant plot of genes shared by MeRIP and RNA-seq group. (**B**) Statistical plot of nine-quadrant plot data in MeRIP and RNA-seq group. (**C**) Nine-quadrant plot of common differential genes shared in early and later periods. (**D**) Nine-quadrant plot of common differential genes shared in peak and later periods. Note: the screening threshold of differential genes/peak is |log2FC| > 1, red and yellow dots indicate that genes and the peak abundance values of m^6^A are consistently upregulated/downregulated, and blue and green dots indicate that genes and the peak abundance values of m^6^A are in opposite direction. Down and up indicate that genes are downregulated in MeRIP group and upregulated in RNA-seq. Up and up indicates genes are all upregulated in MeRIP group and RNA-seq group. Down and down indicates genes are all downregulated in MeRIP group and RNA-seq group. Up and down indicates genes are upregulated in MeRIP group and downregulated in RNA-seq.

To compare the gene expression trends of the two groups, a nine-quadrant diagram was drawn based on using the division standard of the difference multiple. As can be seen from the nine-quadrant diagram, among the 179 DEGs, 150 genes were negatively regulated by m^6^A modification (Figure 5A).

In E vs. M, the expression level of 132 genes was negatively regulated by m^6^A modifications, in which 103 genes were hypomethylated and highly expressed, 2 genes were hypermethylated with low expression, and 27 genes were hypomethylated with low expression. In addition, 17 genes were upregulated in RNA-seq and MeRIP-seq (Figure 5B,C, Supplementary Materials File Table S5).

In P vs. M, 38 genes were hypomethylated and highly expressed, 17 genes were hypomethylated with low expression, and one gene was hypermethylated with low expression. The above 56 genes were all negatively regulated by m^6^A methylation. Additionally, 12 genes were upregulated in both MeRIP-seq and RNA-seq, and were hypermethylated and highly expressed (Figure 5B,D, Supplementary Materials File Table S5).

### 3.7. GO Annotation and KEGG of Common DEGs in MeRIP-seq and RNA-seq

**Figure 6:**
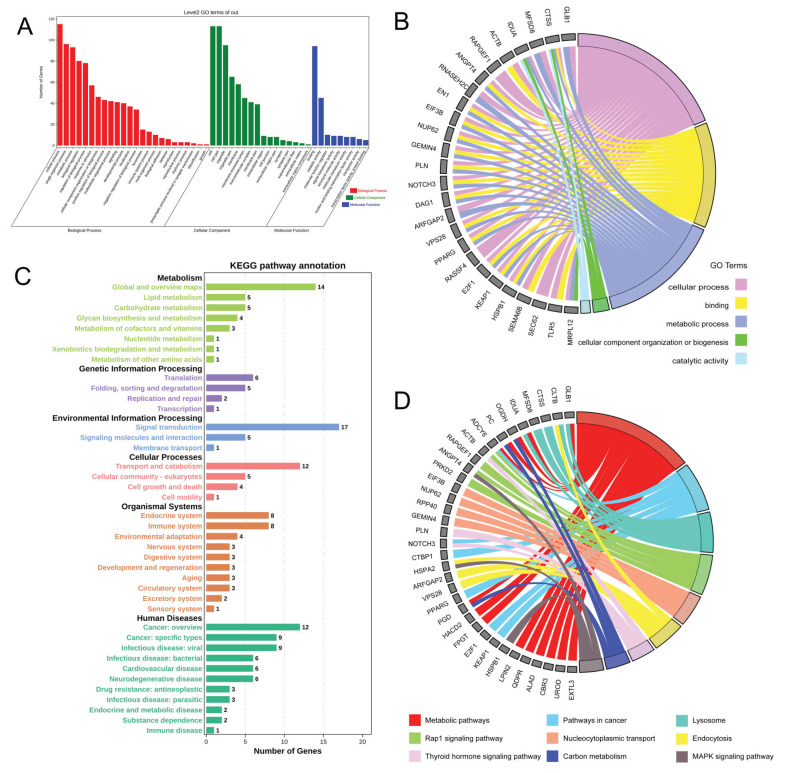
GO annotations and KEGGs for common differential genes in the MeRIP group and the RNA-seq group. (**A**) GO analysis results of common differential genes in the MeRIP group and the RNA-seq group. (**B**) GO annotation of common differential genes related to mammary development and lactation in the MeRIP group and the RNA-seq group. (**C**) KEGG analysis of common differential genes in the MeRIP group and the RNA-seq group. (**D**) KEGG enrichment of common differential genes related to mammary development and lactation in the MeRIP group and the RNA-seq group.

The analysis of GO annotation found, that 179 common DEGs were distributed in 49 secondary GO terms, including 16 cellular components, 24 biological processes, and 9 molecular functions (Supplementary Materials File Table S5). Most genes are concentrated in cellular components, such as cells, cell pairs, organelles, and membranes in biological processes involving cellular processes, single-organism processes, metabolic processes, biological regulation, organization, or biogenesis of cellular components. as well as molecular functions, including binding and catalytic activity. (Figure 6A,B). For the KEGG, 168 pathways were enriched, and some pathways related to mammary gland development and lactation were significantly enriched (Figure 6C,D). For example, 35 genes were annotated in the metabolic pathway, 19 genes were annotated in the Rap1 signaling pathway, and 23 genes were annotated in the MAPK signaling pathway.

### 3.8. Regulatory Network Construction of DMGs in MeRIP-seq and RNA-seq

**Figure 7:**
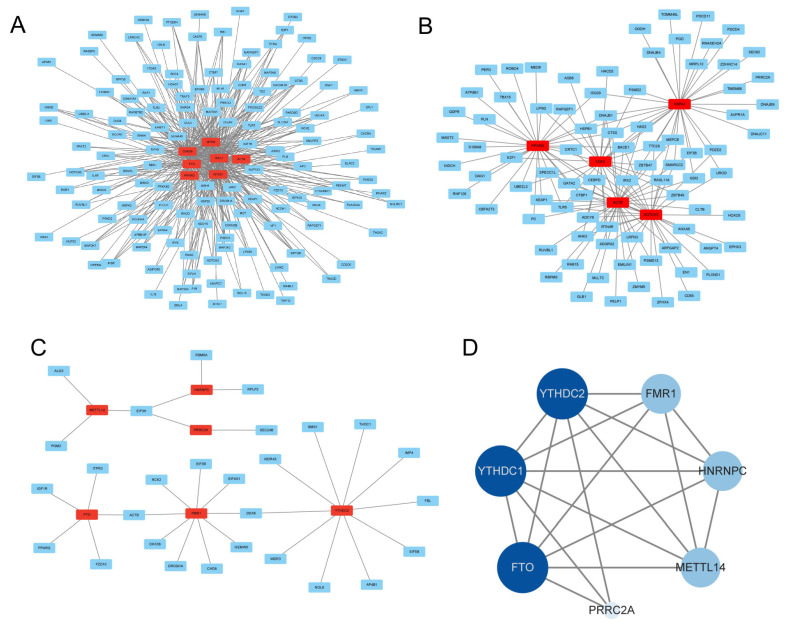
Regulatory network construction in the MeRIP group and the RNA-seq group. (**A**) A regulatory network of seven core differentially methylated genes in the MeRIP group. (**B**) A regulatory network of the five common differential core genes in the MeRIP group and the RNA-seq group. (**C**) A regulatory network constructed with common differential genes and the m^6^A methylases. (**D**) A relationship network of m^6^A differentially methylated enzymes.

Based on GO and KEGG analysis, 343 genes related to mammary gland development and lactation were screened out from the 1539 DMGs obtained in three periods (Supplementary Materials File Table S6), and a regulatory network composed of 7 core genes (*FYN*, *RAC1*, *GSK3B*, *MTOR*, *NFKB1*, *PPARG*, *ACTB*), including 146 nodes and 440 edges, was constructed (Figure 7A). A regulatory network, including 5 core genes (*PPARG*, *HSPA2*, *CDK5*, *ACTB*, *NOTCH3*), composed of 89 nodes and 151 edges, was constructed from the 179 common DEGs in the MeRIP-seq and RNA-seq groups (Figure 7B). In the two networks, *PPARG* and *ACTB* were prevalent, which suggests that they may perform key regulatory functions in mammary gland development and lactation.

Gene interaction analysis was performed on common DEGs and m^6^A methylase in two groups. The interaction network showed that FTO interacted with *ACTB* and *PPARG, PRRC2A* interacted with *SEC24B* and *EIF3H* (Figure 7C), and there were interactions between m^6^A methylase, demethylase, and the reading protein which jointly regualted the m^6^A modification level (Figure 7D). This indicates that m^6^A methylase may upregulate or downregulate the expression level of genes by recognizing their m^6^A modification sites, and may participate in the physiological processes of mammary gland development and lactation.

## 4. Discussion

In this study, we conducted a detailed investigation of m^6^A methylation modification in the mammary gland of dairy goats during different lactation periods based on MeRIP-seq. We demonstrated dynamic, stage specific expression of m6A writers (METTL14), erasers (FTO), and readers (PRRC2A, YTHDC1/2) during dairy goat lactation, identified 1638 differential m^6^A peaks across 1539 genes, and revealed that 83.8% (150/179) of shared DEGs exhibited negative m^6^A-expression correlation. Meanwhile, we found that there were significant differences in the expression levels of methylases, METTL14, PRRC2A, YTHDC1-2, FTO, HNRNPC, and FMR1, in different lactation periods, with PRRC2A and FTO exhibiting the most significance differences (Supplementary Materials File Table S1). It has been found that PRRC2A, as an m^6^A reading protein, can specifically bind to mRNAs with m^6^A modification sites and regulate their stability, playing an important role in cell proliferation [30,31]. Studies on mouse spermatogenesis suggested that PRRC2A is required for correct chromosome arrangement and spindle morphological changes, while it is most likely to activate the spindle assembly checkpoint and lead to metaphase I arrest if the PRRC2A is defective, indicating that a decrease in the expression level of PRRC2A may hinder cell proliferation [32]. As a demethylase, FTO participates in the generation, proliferation and differentiation of adipocytes [33,34]. Studies have shown that overexpression of FTO can significantly increase the triglyceride content in cells, and knock down of FTO can upregulate the m^6^A levels of key cell cycle regulatory factors and autophagy molecules, resulting in a decrease in the expression level of these proteins, thereby inhibiting adipogenesis in 3T3-L1 preadipotasis [35]. Knockdown of Linc-smad7 increases the expression of METTL14, leading to increased methylation and reduced expression level of milk fat synthesis genes *CEBPα* and *PPARγ*, and inhibiting milk fat synthesis [36]. The expression level of the reader protein YTHDC2 is positively correlated with the stage of breast cancer, and knocking down YTHDC2 inhibits the spheroid formation and metastasis ability of breast cancer cells [37]. Reducing METTL3 and METTL14 increases embryo sensitivity to maternal *FMR1* dosage, while FMR1 preferentially binds to mRNA containing the m^6^A-tagged “AGACU” motif to promote its degradation, thereby regulating embryonic development [38].

In order to further elucidate the role of m^6^A modification in the regulation of gene expression, based on the gene expression trends of the two groups, we performed conjoint analysis of data from MeRIP-seq and RNA-seq, and concluded that the expression level of genes with m^6^A modification was higher than that of those without m^6^A modification. m^6^A potentially positively regulates gene expression; in other words, m^6^A modification appears more frequently on genes with a high expression level, while, for genes in which m^6^A has occurred, m^6^A modification also can negatively regulate gene expression [21,39]. Weng et al., (2022) identified a new class of m^6^A reader proteins,—namely, IGF2BP family proteins (IGF2BP1/2/3)—which, in contrast to YTHDF2, can promote the stability and translation of m6A-modified mRNA [40]. In the present study, a total of 179 genes were identified with a differential m^6^A peaks and differential mRNA expression level, which commonly existed in the early peak, and late stages of lactation. Among them, the m^6^A methylation degree of 150 genes was negatively correlated with their expression levels, and 29 common DEGs were also found, indicating that m^6^A methylation positively regulated gene expression (Supplementary Materials File Table S4). For example, from the networks, we obtained information showing that, demethylase FTO interacted with *PPARG*, and they had a positive regulatory trend in the sequencing results. These results showed that highly expressed genes are more likely to bear m^6^A, yet differential m^6^A at specific loci often reduces their expression via decay pathways, and there exists a close relationship with m^6^A modification suggesting that the regulatory direction of m^6^A is gene- and context-specific.

In this study, we identified ten core methylation genes related to mammary gland development and lactation, including *FYN*, *RAC1*, *GSK3B*, *MTOR*, *NFκB1*, *PPARG*, *HSPA2*, *CDK5*, *ACTB*, and *NOTCH3*. Previous studies have indicated that *PPARG* can promote adipocyte proliferation and differentiation, lipid metabolism, and glucose metabolism, and can regulate milk fat synthesis in dairy goats [41,42]. Previous studies have also shown that FTO mediated m^6^A demethylation of *PPARG* and promoted RNA decay of *PPARG* in a YTHDF1-dependent manner, as well as reducing the stability of *PPARG* [43]. In the present study, m^6^A methylation modification negatively regulated *PPARG*, and its expression level was higher during late lactation than during early lactation, indicating that it may be involved in the growth and proliferation of adipocytes for mammary gland remodeling during late lactation. Previous studies have shown that METTL3 can activate the Notch pathway by regulating its target gene *NOTCH3* [44]. Notch signaling is widely involved in cell biological processes, including cell proliferation, apoptosis and the epithelial–mesenchymal transition. It is essential for mammary gland development and normal function [45]. Studies have indicated that changing the expression of *FYN* affects the differentiation, proliferation, migration and invasion of MGECs [46,47], and YTHDF2 can enhance the stability of *FYN* transcripts [48]. The mTOR signaling pathway is activated by amino acids, insulin, and growth factors, which allow it to participate in the regulation of the synthesis of milk protein and fatty acid [49], the proliferation and differentiation of MGECs, as well as mammary gland development [50]. *NFκB1*, as a transcription factor, is involved in inflammation and immune responses, and controlled cell differentiation and survival by regulating the expression of *mTOR* in milk synthesis and the proliferation of MGECs [51,52]. However, m^6^A modification is closely related to the mTOR signaling pathway, especially for METTL3 and FTO, which comprehensively regulate various genes in the pathway [22,53]. These genes play extensive and important roles in breast development and lactation regulation by participating in metabolic pathways and signal transduction, ensuring the normal development and proper functions of mammary glands. In addition, other core genes discovered in this study, such as *Rac1*, *GSK3B*, *ACTB*, *HSPA2,* and *CDK5*, are also widely involved in the biological regulation of mammary gland development and lactation by regulating the proliferation, differentiation and cell cycle of MGECs, mammary gland remodeling, milk secretion and synthesis, etc. For example, studies have shown that *RAC1* can remove apoptotic cells and excess milk during the later stages of lactation and eliminate inflammation to facilitate mammary gland remodeling [54]. *GSK3B* participates in various signaling pathways–such as Wnt, PI3K-AKT, mTOR, Hedgehog, and Notch–to regulate the cell cycle, cell proliferation, differentiation, migration, and milk synthesis, as well as maintaining normal mammary gland functions [55]. *ACTB* is an important structural protein of the cytoskeleton, which can regulate cell growth, migration, and division. The absence of Rac1 signaling results in mammary gland involution and cell death by epithelial phagocytes [56]. The overexpression and inhibition of *HSPA2* and *CDK5* are related to the proliferation, apoptosis, and migration of breast cancer cells. Meanwhile, Cdk5 is essential for brain development during embryogenesis, and provides a pleiotropic contribution in regulating neuronal actin cytoskeletal remodeling [57,58].

Based on the information presented above, it can be inferred that, by altering global m^6^A levels, methylases may be closely related to characteristics of the development and morphology of cells, and may participate in the proliferation, differentiation, and apoptosis of MGECs; the synthesis of milk fat; the differentiation of adipocytes; and immune inflammation, among other processes. We speculate that m^6^A methylation modification affects mammary gland development and lactation by regulating the expression of these core genes, which are typically involved in key processes such as mammary gland cell proliferation, differentiation, and apoptosis, as well as milk synthesis and secretion. Further experiments are needed to confirm these regulatory relationships and the corresponding effects on MGECs in vivo or in vitro.

## 5. Conclusions

In this study we employed an integrated analysis of MeRIP-seq and RNA-seq to investigate the expression patterns and functional roles of methylation peaks and associated genes in the mammary gland tissue of dairy goats across different lactation stages. Ten hub genes (*FYN*, *RAC1*, *GSK3B*, *MTOR*, *NFκB1*, *PPARG*, *HSPA2*, *CDK5*, *ACTB*, and *NOTCH3*) and five methylases (METTL14, FMR1, PRRC2A, YTHDC1-2, and FTO) were scanned and identified. The obtained data suggests that these genes may perform regulatory roles in mammary gland development and milk synthesis. This study provides preliminary insights into the epigenetic modification profile of m^6^A in full the lactation cycle of dairy goats, contributing valuable knowledge to improve our understanding of mammary gland physiology in ruminants.

## Figures and Tables

**Table 1:** Quality analysis and reference genome comparison of sequencing data.

Sample	Raw Data	Clean Data and Base Quality			Reference Genome Mapping		
		Clean Data	Q20 (%)	Q30 (%)	Unique_Mapped_Reads	Multiple_Mapped_Reads	All Mapped Reads
E1-IN	49336232	48847722 (99.01%)	6989937247 (97.26%)	6613256954 (92.02%)	41781542 (85.75%)	3549962 (7.29%)	45331504 (93.04%)
E1-IP	47274568	45972812 (97.25%)	2021227037 (92.21%)	1871690851 (85.39%)	25455216 (55.42%)	9434574 (20.54%)	34889790 (75.96%)
E2-IN	52823280	52304560 (99.02%)	7497513916 (97.32%)	7095665407 (92.10%)	45085065 (86.34%)	4680842 (8.96%)	49765907 (95.31%)
E2-IP	50759632	49362212 (97.25%)	2216090699 (92.50%)	2054835901 (85.77%)	30472303 (61.76%)	8603775 (17.44%)	39076078 (79.20%)
E3-IN	64813138	64543396 (99.58%)	9254754450 (97.39%)	8792123099 (92.53%)	55296223 (85.92%)	6533475 (10.15%)	61829698 (96.07%)
E3-IP	58311116	57006772 (97.76%)	2373500143 (93.15%)	2216974301 (87.00%)	36019943 (63.23%)	10062207 (17.66%)	46082150 (80.89%)
M1-IN	45990328	45873914 (99.75%)	6676884375 (97.26%)	6360487594 (92.65%)	41528790 (90.58%)	2293689 (5.00%)	43822479 (95.58%)
M1-IP	60920318	59851776 (98.25%)	4620013058 (98.50%)	4493473372 (95.80%)	46720976 (78.13%)	6314407 (10.56%)	53035383 (88.69%)
M2-IN	45138384	45022574 (99.74%)	6559955218 (97.40%)	6261243385 (92.96%)	38979574 (86.71%)	1889772 (4.20%)	40869346 (90.92%)
M2-IP	59322628	56784348 (95.72%)	3227223883 (98.05%)	3115696572 (94.66%)	31123475 (54.89%)	7857644 (13.86%)	38981119 (68.74%)
M3-IN	52372706	52283468 (99.83%)	7644172658 (97.65%)	7296392557 (93.21%)	47941023 (91.72%)	2080862 (3.98%)	50021885 (95.70%)
M3-IP	62105882	60811202 (97.92%)	4300057659 (98.25%)	4167031814 (95.21%)	47128789 (77.64%)	8972234 (14.78%)	56101023 (92.42%)
P1-IN	50993434	50610932 (99.25%)	7295972140 (97.32%)	6901424197 (92.06%)	43640296 (86.38%)	4544886 (9.00%)	48185182 (95.37%)
P1-IP	44199300	43162816 (97.65%)	1688812739 (93.05%)	1577322072 (86.91%)	27756879 (64.34%)	6412478 (14.86%)	34169357 (79.20%)
P2-IN	49823380	49382116 (99.11%)	7017856509 (97.28%)	6651148714 (92.20%)	43060635 (87.49%)	3391325 (6.89%)	46451960 (94.39%)
P2-IP	49680806	48674586 (97.97%)	2206924336 (93.49%)	2056514036 (87.12%)	29752521 (61.17%)	9099850 (18.71%)	38852371 (79.88%)
P3-IN	59977268	59518652 (99.24%)	8522800666 (97.60%)	8108542674 (92.85%)	50915336 (85.69%)	6397019 (10.77%)	57312355 (96.45%)
P3-IP	46226638	45333466 (98.07%)	2087434412 (93.51%)	1947410259 (87.24%)	31992607 (70.60%)	5985301 (13.21%)	37977908 (83.81%)

**Table 2:** Mainly enriched motif sequences in different lactation periods.

Period	DRACH	Motif	Motif Number	RRACH	Motif	Motif Number
E	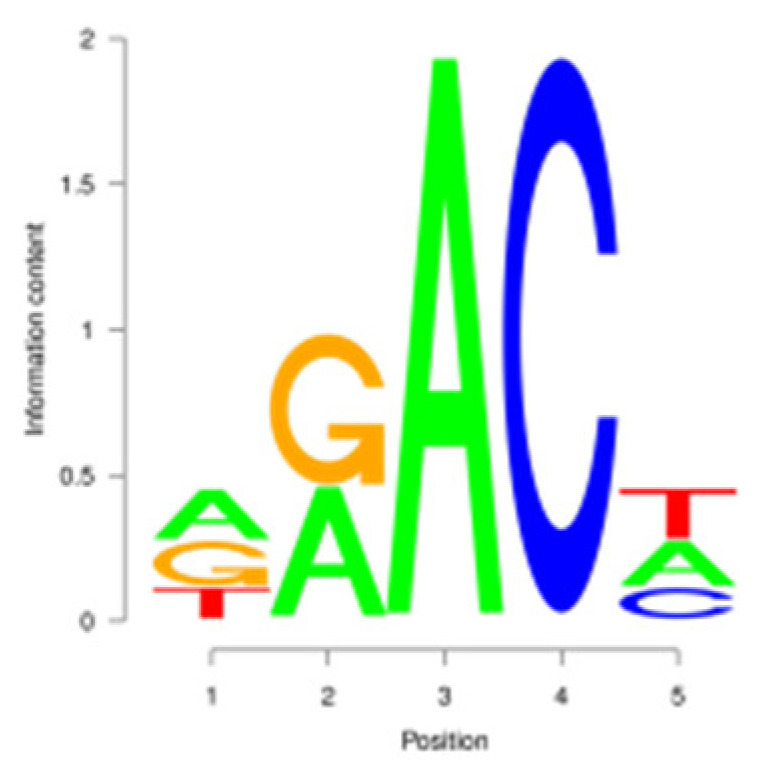	AAACA	1258 (9.05%)	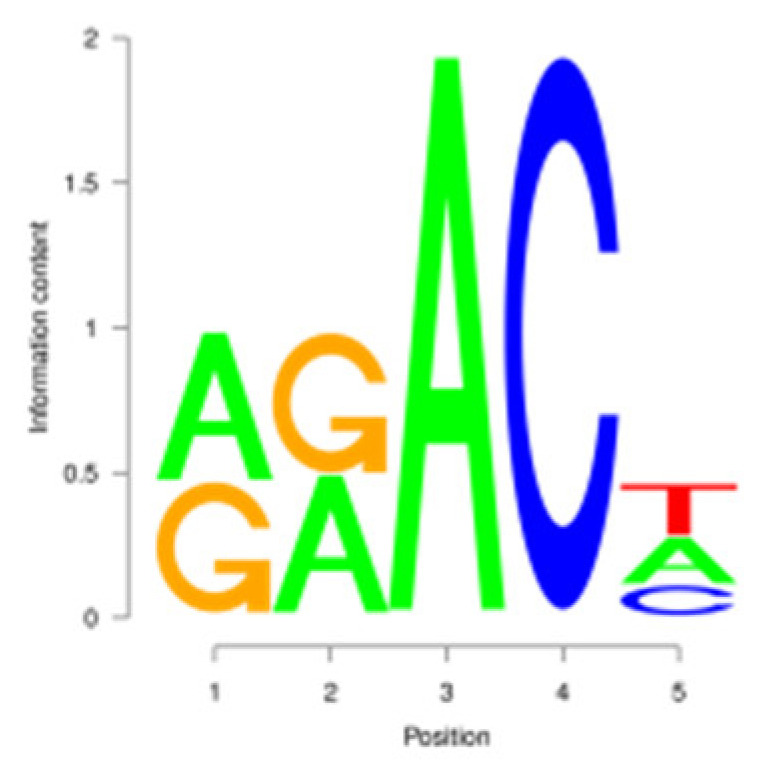	AAACA	1281 (11.93%)
P	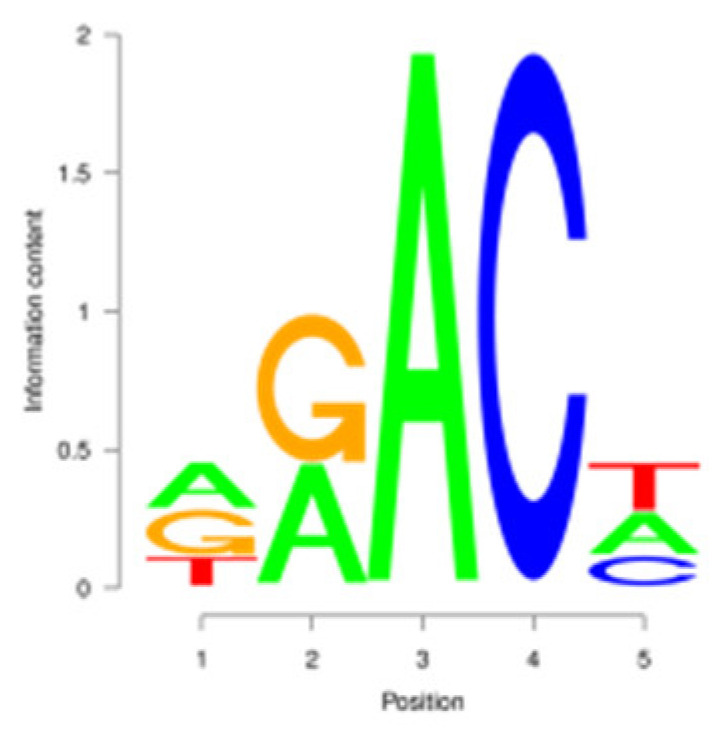	GGACT	709 (9.31%)	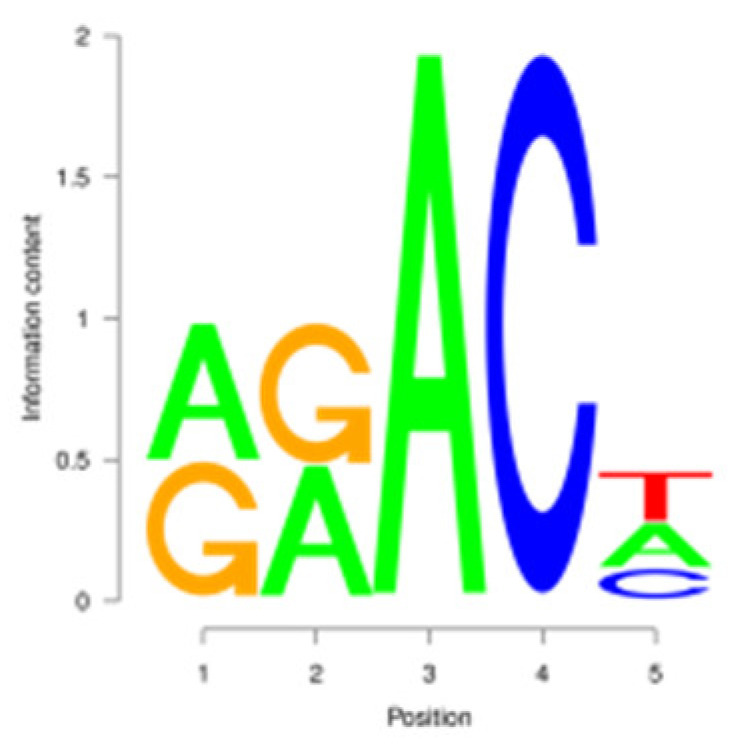	GGACT	732 (12.35%)
M	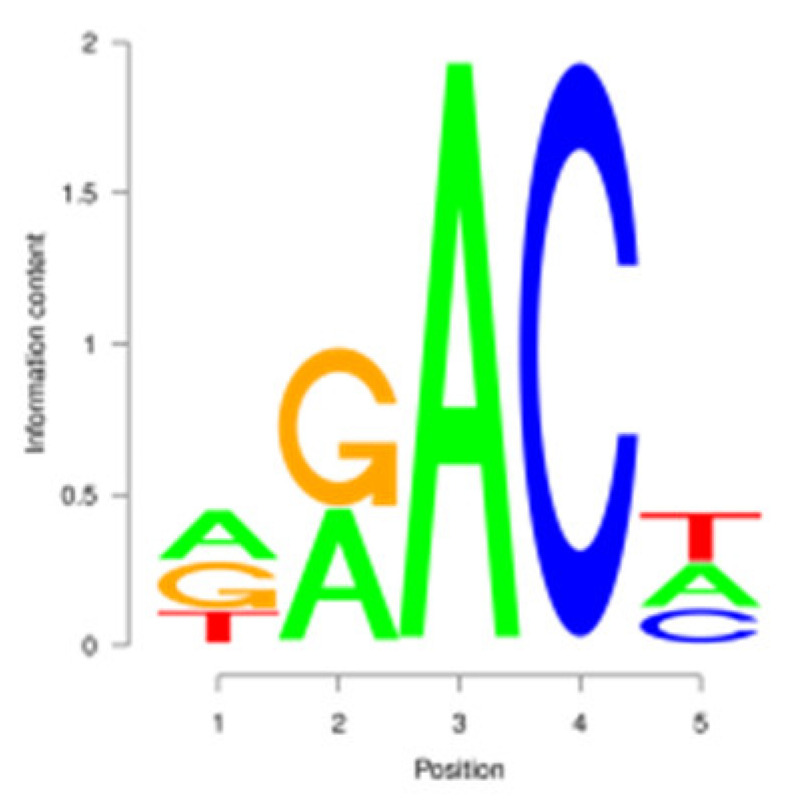	AAACA	218 (8.92%)	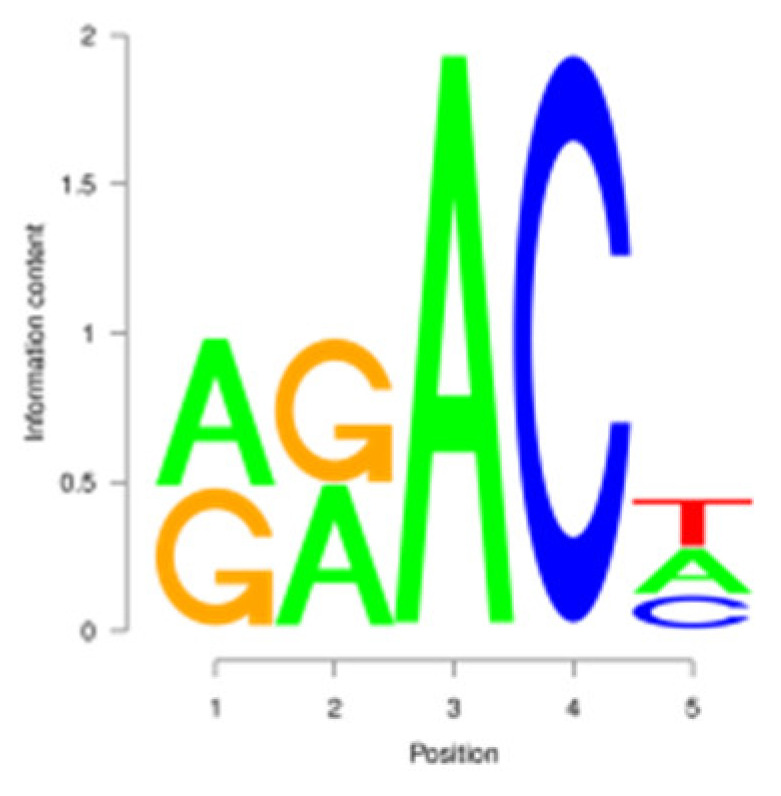	AAACA	222 (11.76%)

Note: Sequence characteristics of m^6^A modification: RRACH, DRACH (D = G/A/U, R = G/A, H = A/U/C).

## Data Availability

The data that support the findings of this study are available in the tables and Supplementary Materials. The raw sequencing data were deposited in the Sequence Read Archive (PRJNA1193216).

## Supplementary Materials

The following supporting information can be downloaded at: https://www.mdpi.com/article/10.3390/ani15192775/s1, Table S1. m6A methylase information. Table S2. All sample peak notes. Table S3. Information of differentially methylated peaks. Table S4. RNA-seq differential genes. Table S5. DEGs shared by MeRIP-seq and RNA-seq. Table S6. Genes associated with development and lactation. Table S7. Ethical approval.
