# Supplementary material for: Epigenetic Effects and Potential Contributions of m6A Modification to Mammary Gland Development and Lactation of Dairy Goats Explored via MeRIP-seq
Source: Animals (Basel). 2025 Sep 23;15(19):2775. doi: 10.3390/ani15192775 (PMC12523381; doi:10.3390/ani15192775)
Supplement: Supplementary file 1 [file animals-15-02775-s001.zip › Table S7. Ethical approval.pdf]

实验动物使用许可

(SDAUA-2021-130)

根据《山东农业大学实验动物管理条例》，山东农业大学实验动物管理与使用委员会经过认真审查并讨论研究决定：批准我院动物生态保护生物学实验室自即日起使用山羊组织进行相关实验。要求该实验室严格《按照山东农业大学实验动物管理条例》规定，所有操作实验动物的人员(包括教师、实验员、博士后、博士生和硕士生)都必须事先经过培训，才能使用实验动物。实验动物必须在动物科技学院下属的山东农业大学实验动物中心监督下，于严格控温、控光的动物舍内进行正常饲养，操作和处死动物要严格遵守动物福利的原则。

特此许可。

动物科技学院

2021 年 4 月 10 日
